# Nucleophilic Substitution at Tricoordinate Sulfur—Alkaline Hydrolysis of Optically Active Dialkoxysulfonium Salts: Stereochemistry, Mechanism and Reaction Energetics

**DOI:** 10.3390/molecules27238212

**Published:** 2022-11-25

**Authors:** Marian Mikołajczyk, Bogdan Bujnicki, Józef Drabowicz, Marek Cypryk

**Affiliations:** 1 Department of Organic Chemistry, Centre of Molecular and Macromolecular Studies, Sienkiewicza 112, 90-363 Łódź, Poland; 2 Department of Structural Chemistry, Centre of Molecular and Macromolecular Studies, Sienkiewicza 112, 90-363 Łódź, Poland

**Keywords:** dialkoxysulfonium salts, alkaline hydrolysis, stereochemistry, addition-elimination (A–E) mechanism, pseudorotation, DFT calculations

## Abstract

Optically active dialkoxyisopropylsulfonium salts were obtained by methylation (ethylation) of optically active alkyl isopropanesulfinates using methyl (ethyl) trifluoromethanesulfonate. Alkaline hydrolysis of a series of methoxy(alkoxy)sulfonium salts afforded the two sulfinate products methyl isopropanesulfinate and alkyl isopropanesulfinate, both formed with a slightly prevailing inversion of configuration at the sulfur atom. DFT calculations revealed that this substitution reaction proceeded stepwise according to an addition-elimination (A–E) mechanism involving the formation of high tetracoordinate S^IV^ sulfurane intermediates. In addition, the DFT calculations showed that recombination of the hydroxy anion with the methoxy(alkoxy)sulfonium cation—leading to the parallel formation of the two most stable primary sulfuranes, with the hydroxy and alkoxy groups in apical positions and their direct decomposition—is the most energetically favorable pathway.

## 1. Introduction

The majority of reactions with heterorganic compounds (H=P, S, Si, Se…) are typical bimolecular nucleophilic substitution reactions of the S_N_2-H type, occurring at heteroatom centers. The stereochemical course and mechanisms of these reactions have been a subject of extensive studies in the second half of the past century. It has been found that in the overwhelming majority of cases, they proceed with inversion of configuration at the stereogenic phosphorus and sulfur atoms. Although the observation of inversion may be rationalized by the concerted S_N_2-H mechanism via a transition state, an alternative addition-elimination mechanism (A–E) involving the formation of trigonal bypiramidal intermediates (P^V^-phosphorane and S^IV^-sulfurane) has also been considered.

**Scheme 1:**
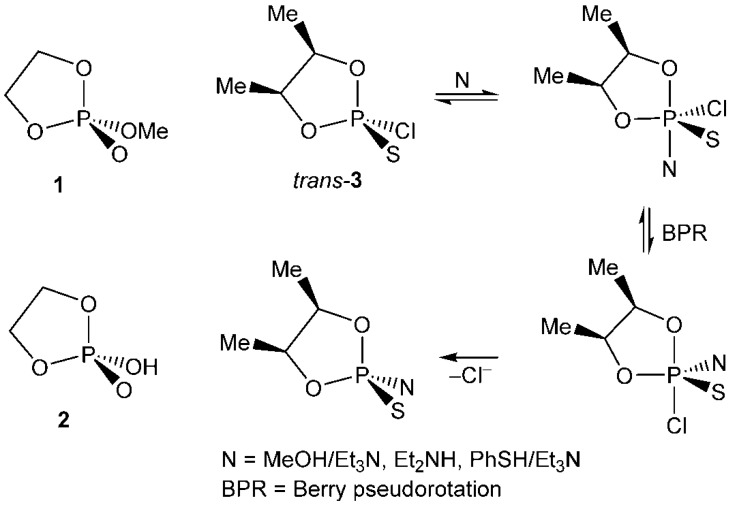
Stereochemical course of substitution at the phosphorus in cyclic five-membered thiophosphoryl chlorides.

In 1968, Westheimer [1] provided convincing arguments for the operation of the A–E mechanism in the acid-catalyzed hydrolysis of methyl ethylene phosphate **1** (2-methoxy-1,3,2-dioxaphospholan-2-one) and in the ^16^O–^18^O exchange reaction in hydrogen ethylene phosphate **2** (2-hydroxy-1,3,2-dioxaphospholan-2-one). A few years later, we provided experimental stereochemical evidence in support of the Westheimer concept of a substitution at P in cyclic five-membered phosphates (P^V^-intermediate and its pseudorotation) as it was found that the geometrical cis*-* and trans*-*isomers of 2-chloro-4,5-dimethyl-1,3,2-dioxaphospholan-2-thione react with nucleophilic reagents, with retention of their configuration at phosphorus [2]—see Scheme 1.

**Figure 1:**
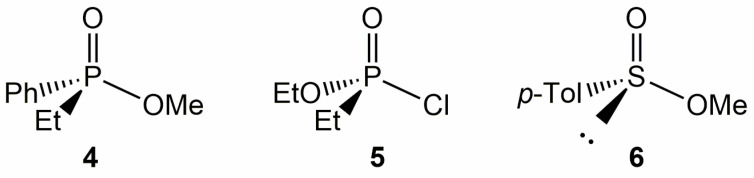
Structures of model compounds used in our studies on identity reactions.

From that time, the other nucleophilic substitution reactions occurring with retention of configuration at the heteroatom centers (see, for example, [3,4]) have been rationalized in terms of the Westheimer’s concept and the addition-elimination mechanism, involving the formation of high-coordinate intermediates and their pseudorotation. However, it should be pointed out here that these transiently formed intermediates have not been isolated or detected by spectroscopic methods due to their instability. Moreover, in this early period of studies on nucleophilic substitution reactions at heteroatoms, it was not possible to make a distinction between the S_N_2-H and the A–E mechanisms. A solution of this fundamental question was especially interesting for the identity substitution reactions at the chiral phosphorus and sulfur atoms. Taking advantage of the results of theoretical studies on substitution reactions at heteroatoms (P, S, Si) in simple achiral model compounds [5,6,7,8], we performed [9] the DFT calculations for the three symmetrical displacement reactions in the optically active phosphorus and sulfur compounds, as shown below (Figure 1).

The kinetic measurements provided direct evidence that each elementary substitution step in these reactions occurs with a full inversion of configuration at the phosphorus and sulfur [10,11,12]. Now it has been found [9] that the methoxy–methoxy exchange reactions in phosphinate **4** and sulfinate **6** proceed in a stepwise fashion via the corresponding P^V^-phosphorane and S^IV^-sulfurane, respectively. The latter intermediates with the two methoxy groups in apical positions are the most stable, do not undergo pseudorotation and decompose directly to the starting compounds **4** and **6**, but with opposite configurations at P and S. On the other hand, the chloride–chloride exchange at phosphorus in **5** is a typical concerted S_N_2-P process.

**Figure 2:**
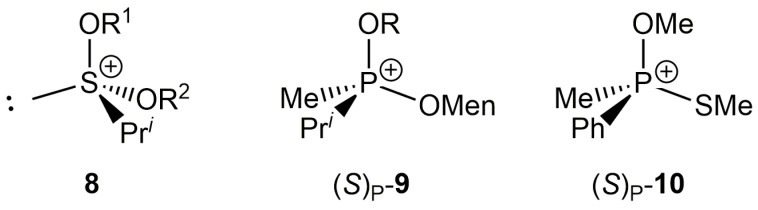
Structures of dialkoxysulfonium and phosphonium salts used in the present and in earlier studies on nucleophilic substitution at heteroatoms (anions omitted).

In the hope of gaining further insight into the mechanism of displacement reactions at the sulfur atom, we decided to study the stereochemistry-mechanism relationship in the alkaline hydrolysis of optically active dialkoxysulfonium salts **8**. This sulfur structure was selected for our study for two reasons. First, it contains two potential leaving groups bonded to sulfur and their leaving ability and apicophilicity are almost the same. Secondly, it was interesting to compare our results on the hydrolysis of **8** with those reported by DeBruin and Mislow [13,14] on the alkaline hydrolysis of diastereomeric dialkoxyphosphonium salts **9** and alkoxy(alkylthio)phosphonium salt **10** (Figure 2)**.**

In the present paper, we report the complete results of our investigations on the sulfonium salts **8**, including the asymmetric synthesis of enantiomerically enriched isopropanesulfinic acid esters and their conversion to the corresponding dialkoxysulfonium salts **8**; then, the stereochemical course of the alkaline hydrolysis of the salts **8** and the DFT studies aimed at the univocal determination of the mechanism of the investigated title reaction.

## 2. Results and Discussion

### 2.1. Synthesis of Optically Active Dialkoxysulfonium Salts ***8***

**Scheme 2:**
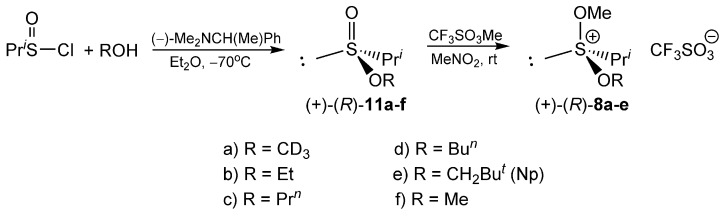
Synthesis of optically active dialkoxysulfonium salts **8**.

Racemic dialkoxysulfonium salts were obtained for the first time by Kobayashi and coworkers [15,16] in the reaction of sulfinic acid esters with strong alkylating reagents. The authors found that only alkanesulfinates—in contrast to arenesulfinates—form the corresponding dialkoxysulfonium salts, which are relatively stable in a solution of nitromethane. Having this in mind, we decided to prepare in the first step a series of optically active alkyl isopropanesulfinates **11** as precursors of the sulfonium salts **8**. The sulfinates **11** were easily obtained according to our method [17], which in this case consisted in the asymmetric condensation of racemic isopropanesulfinyl chloride with achiral alcohols in the presence of (−)-*N*,*N*-dimethyl-α-phenylethylamine as an asymmetric reagent (see Scheme 2). All the dextrorotatory sulfinates **11a–f** so-formed were enriched in the (*R*)-enantiomers and their optical purities were in the range of 10–27% (see Table 1).

A subsequent reaction of the sulfinates **11a–e** with methyl trifluoromethanesulfonate, carried out in nitromethane, afforded the required optically active alkoxy(methoxy)isopropylsulfonium salts **8a–e**. As the methylation takes place at the sulfinyl oxygen atom in the sulfinates **11**, the configuration at the chiral sulfur atom in the sulfonium salts **8** remains unchanged and is also defined as *R* in accordance with the Cahn, Ingold and Prelog rules [18]. The values for the optical rotation of the isolated sulfonium salts **8a–e** were very low expressing, indicating that their intrinsic asymmetry was extremely small. Selected data on alkyl sulfinates **11**, sulfonium salts **8** and sulfinates **11**, obtained after their alkaline hydrolysis, are summarized in Table 1.

### 2.2. Stereochemical Course of the Alkaline Hydrolysis of Dialkoxysulfonium Salts ***8***

**Scheme 3:**
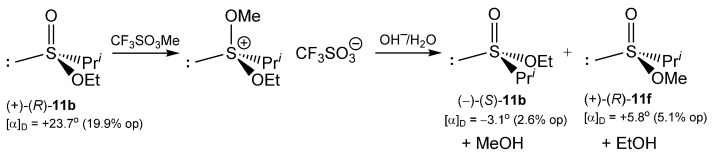
Stereochemical outcome of the alkaline hydrolysis of sulfonium salt (+)-(*R*)-**8b**.

Due to their limited stability, the freshly prepared and isolated salts **8** were promptly dissolved in water and treated with a diluted solution of sodium hydroxide at room temperature. The hydrolysis reaction was terminated when the reaction solution became basic. The hydrolysis products (usually two sulfinates **11**) obtained were separated and characterized. The outcome of the alkaline hydrolysis of (+)-(*R*)-ethoxy(methoxy)isopropylsulfonium trifluoromethanesulfonate **8b**, shown in Scheme 3, best illustrates the experiments that were also performed with the other sulfonium salts **8**.

Thus, the (+)-(*R*)-ethoxy(methoxy)isopropylsulfonium salt (**8b**) prepared by methylation of (+)-(*R*)-sulfinate **11b** gave upon alkaline hydrolysis two sulfinates: ethyl isopropanesulfinate (−)-(*S*)-**11b** and methyl isopropanesulfinate (+)-(*R*)-**11f**. The first sulfinate, isolated from a mixture by preparative gas chromatography, was formed with an inverted configuration at the sulfur with respect to the starting configuration—indicating that hydrolysis of the sulfonium salt (+)-(*R*)-**8b** occurred with inversion at the sulfur atom. The second hydrolysis product, dextrorotatory methyl isopropanesulfinate **11f**, has the same absolute configuration (*R*) as other alkyl isopropanesulfinates **11** produced by our asymmetric synthesis. A comparison of the chirality at sulfur in the starting sulfonium salt **8b** with that of the sulfinate (+)-(*R*)-**11f** provides evidence that the latter ester is also formed with the inversion of its configuration at the sulfur. However, it should be noted that both esters **11b** and **11f**, isolated after alkaline hydrolysis and separated by preparative gas chromatography, are highly racemized.

**Scheme 4:**
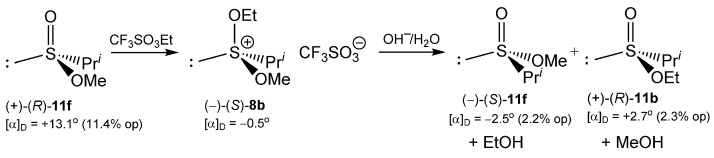
Stereochemical outcome of the alkaline hydrolysis of sulfonium salt (−)-(*S*)**-8b**.

To check whether the applied experimental procedure and the results of the alkaline hydrolysis of the sulfonium salt (+)-(*R*)-**8b** are reproducible, the (−)-(*S*) enantiomer of **8b** was prepared and hydrolyzed under the same conditions. It is interesting to note that the sulfonium salt (−)-(*S*)**-8b** was obtained by the ethylation of methyl isopropanesulfinate (+)-(*R*)-**11f** using ethyl trifluoromethanesulfonate. The results of the alkaline hydrolysis of the salt (−)-(*S*)**-8b** are shown in Scheme 4.

An inspection of the results depicted above revealed that (a) both sulfinates **11f** and **11b**, being the hydrolysis products of the sulfonium salt (−)-(*S*)**-8b**, are formed with a prevailing inversion of their configuration, (b) the alkaline hydrolysis of the salt (−)-(*S*)**-8b** is accompanied by other side processes responsible for a great extent of racemization of the sulfinates **11b** and **11f** and (c) the experimental procedure for the alkaline hydrolysis of the **8** salts and the isolation of the **11** sulfinates is reproducible.

**Scheme 5:**
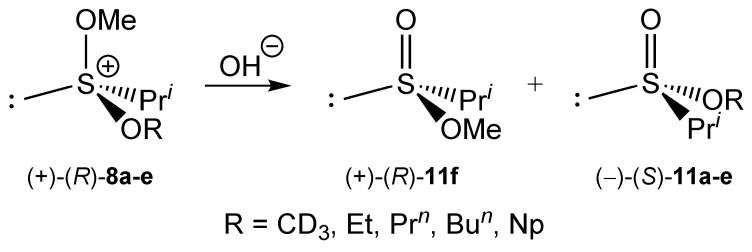
Stereochemical outcome of the alkaline hydrolysis of sulfonium salts **8a–e**.

To get more information on the effect of an alkoxy substituent on the course of the investigated reaction in particular, a series of optically active methoxy(alkoxy)isopropylsulfonium salts (+)-(*R*)-**8a–e** was subjected to mild alkaline hydrolysis. In accordance with the results described above on the hydrolysis of the salt (+)-(*R*)-**8b**, all the reactions performed—with one exception—afforded sulfinate esters **11b–e** with the defined stereochemistry depicted in Scheme 5.

It was found that the proportions of the two sulfinates formed in the above reactions varied and were dependent on the size of the alkoxy substituent. The content of methyl sulfinate **11f** in the isolated mixture of both the esters that was formed was determined by ^1^H NMR spectroscopy and gas chromatography. Table 2 shows the results.

It turned out that with an increase in the steric bulk of the alkoxy group in sulfonium salts **8**, the formation of methyl sulfinate **11f** gradually decreased. In the case of the alkaline hydrolysis of methoxy(neopentoxy)sulfonium salt **8e**, methyl sulfinate **11f** was not formed. A small amount of methyl sulfinate **11f** that formed upon the hydrolysis of the sulfonium salts **8c** and **8d** was the main reason that our attempts to obtain this ester in a pure state by preparative gas chromatography were unsuccessful. Moreover, it is reasonable to assume that the other methyl sulfinates **11f** are formed in these hydrolysis reactions with an inversion of their configuration and that are highly or completely racemized due to the side reactions discussed below. As sulfinates **11b–e** were the major hydrolysis products of salts **8b–e**, they were obtained in a pure state by preparative gas chromatography—although with a low yield. All these esters were highly racemized but formed with a predominant inversion of their configuration at the sulfur.

### 2.3. DFT Study on the Mechanism-Stereochemistry Relationship in the Alkaline Hydrolysis of Sulfonium Salts ***8*** and a Question of the Racemization of Sulfinate Products

To rationalize the stereochemical results and other observations on the alkaline hydrolysis of dialkoxysulfonium salts **8**, it is necessary to first solve the question of the mechanism of this reaction. The hydrolysis of the sulfonium ion was performed in water. The solvation effect was estimated within an SCRF-CPCM approximation, in which the solvent was treated as the continuous medium, characterized by its dielectric constant.

**Scheme 6:**
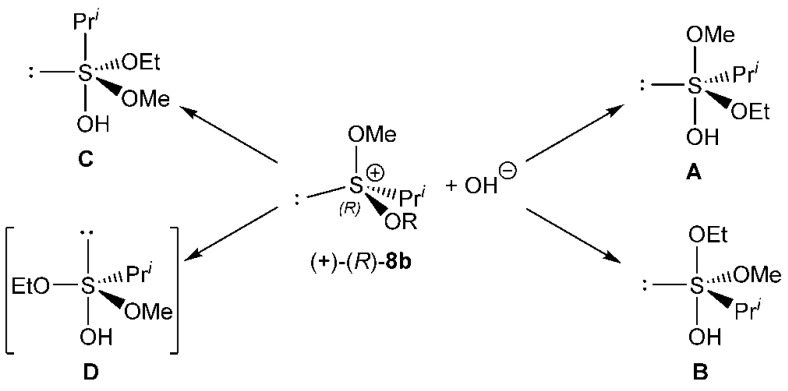
Four possible S^IV^-sulfurane structures formed by the addition of a hydroxy ion to (+)-(*R*)-**8b**.

**Scheme 7:**
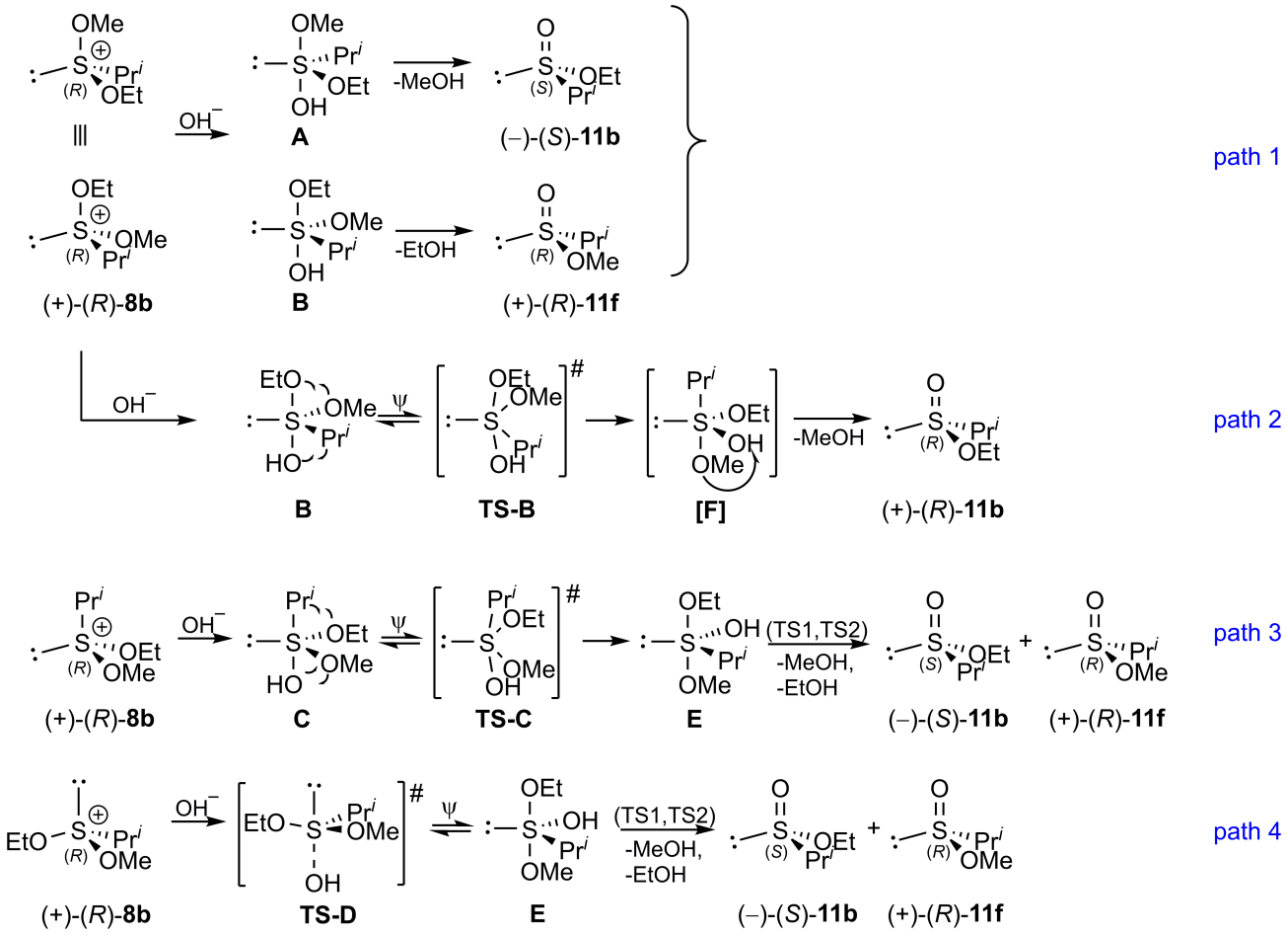
Possible pathways for the replacement of the methoxy and ethoxy group in sulfonium cation **8**.

DFT calculations for the hydrolysis of the dialkoxysulfonium salt excluded the concerted one-step S_N_2-type substitution and confirmed the (A–E) mechanism. The attack of the hydroxyl ion on the four walls of the sulfonium trigonal pyramid led to the tetracoordinate sulfurane structures **A**, **B** and **C**, in which their relative energies were calculated (Scheme 6). Structure **D** could not be located as during the geometry optimization, the system transforms (via a spontaneous pseudorotation) into the more stable structure **E**, with both alkoxy groups occupying apical positions (see path 4 in Scheme 7 and the corresponding discussion below).

The Gibbs free energy of the alkaline hydrolysis of ethoxymethoxysulfonium salt (Equation (1)) is ΔG_r_ = −63.4 kcal/mol. The energy of the ion recombination is partially responsible for such a great negative value. The formation of tetracoordinate sulfurane proceeds practically without an energy barrier (the barriers associated with the rearrangement of solvent molecules cannot be estimated within the SCRF approximation).

(EtO)iPrSOMe^(+)^ + OH^(−)^ → [(EtO)iPrS(OMe)OH] → (MeO)iPrS=O + EtOH (1)

We analyzed by DFT calculation the possible substitution routes, which are presented in Scheme 7.

**Scheme 8:**
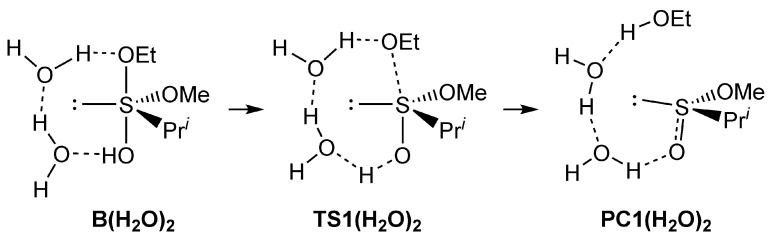
Structures of the reactive complex **B(H_2_O)_2_**, transition state **TS1(H_2_O)_2_**, and product complex **PC1(H_2_O)_2_** of the (H_2_O)_2_-mediated substitution of OEt by OH in (+)-(*R*)**-8b**; the substitution of OMe by OH proceeds analogously.

Path 1 is the simplest route, in which the hydroxyl ion approaches the sulfonium cation from the opposite side to one of the alkoxy groups, forming low energy sulfuranes **A** or **B** in which two electronegative substituents—OH and OR—occupy the apical positions. In line with the commonly accepted substitution scheme, the departure of the apical group should be facilitated. This scheme implies an inversion of the configuration at the central sulfur atom, leading to (−)-(*S*)-**11b** and (+)-(*R*)-**11f** as a result of the evolution of MeOH and EtOH, respectively. This process requires the transfer of a proton from the hydroxyl group to the leaving alkoxyl group. Such a transfer is not possible directly. However, in a water solution, it can be easily mediated by solvent molecules. To prove this, we calculated the proton transfer through a bridge of two water molecules. The process, resulting in the substitution of the ethoxy group, is illustrated in Scheme 8. The substitution of the methoxy group proceeds analogously; the calculations confirmed that this process proceeds virtually without an energy barrier.

**Scheme 9:**
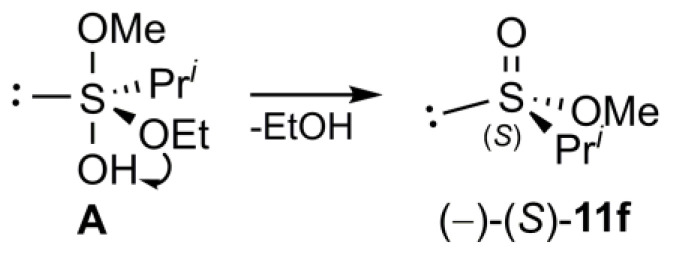
The departure of the ethoxy group from the equatorial position in sulfurane (**A**); the process of the elimination of methanol from the equatorial position in (**B**) proceeds analogously.

**Figure 3:**
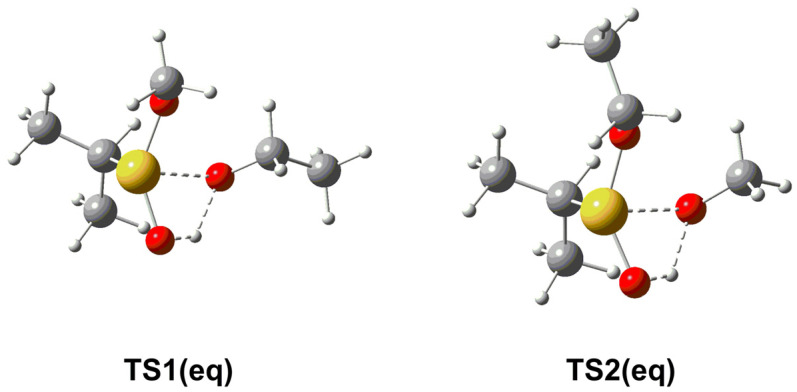
Transition states for the equatorial departure of the alkoxy group, assisted by the apical OH group; color meaning: sulfur—yellow, oxygen—red, carbon—grey, hydrogen—white.

The alternative possibility one could imagine is the departure of the alkoxy group from the equatorial position. In such a case the proton transfer is possible, but this reaction is associated with a rather high energy barrier (transition states **TS1(eq)** and **TS2(eq)** for the substitution of OEt and OMe, respectively); see Scheme 9 and Figure 3.

**Figure 4:**
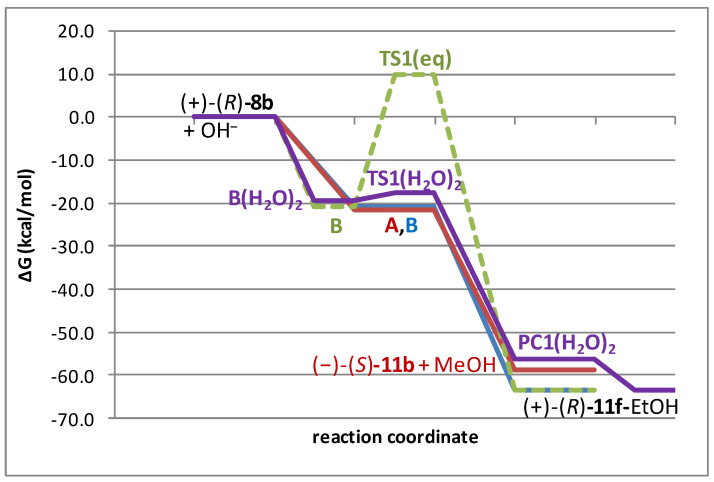
Gibbs free energy profile for reaction path 1; continuous lines correspond to the departure from the apical position of EtOH (blue) and MeOH (red), respectively; the dashed line corresponds to the departure of EtOH from the equatorial position (green) and the violet line corresponds to the substitution of EtOH mediated by (H_2_O)_2_; descriptions of stationary points are in the color of the corresponding line for clarity.

The free energy profiles for the discussed variants of reaction path 1 are presented in Figure 4. Note that the barriers associated with changes in solvation cannot be easily calculated, but they are expected to be very low; thus, the main reaction profile is shown as virtually barrierless. Some pathways involving the formation of MeOH are omitted for clarity as they are almost identical to those involving the formation of EtOH. The sum of the free energies of the substrates ((+)-(*R*)-**8b** and OH^−^, as well as (H_2_O)_2_ in the case of water dimer-mediated substitution), was taken as zero.

Path 2 discusses the possibility of the Berry pseudorotation (ψ) of **A** and **B** to give sulfurane with Pr*^i^* and OR (R = Me, Et) groups in apical positions (**F)**. However, structure **F** could not be found, as in the pseudorotational transition states (**TS-A** and **TS-B**), a spontaneous proton transfer from the OH to the equatorial alkoxy group occurs, resulting in the departure of the ROH and the formation of the corresponding sulfinate **11**. The products of the transformation of **A** and **B** according to path 2 have opposite configurations to those produced in path 1 and, if created, this process would lead to complete racemization—which is contrary to the experimental results. The calculated energy profile for path 2 also shows that the free energy barrier of this transformation is relatively high, making this reaction pathway rather unlikely.

**Scheme 10:**
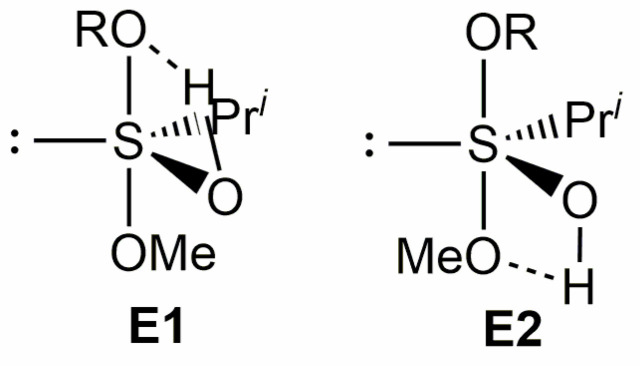
Conformers of the most stable sulfurane **E**.

**Figure 5:**
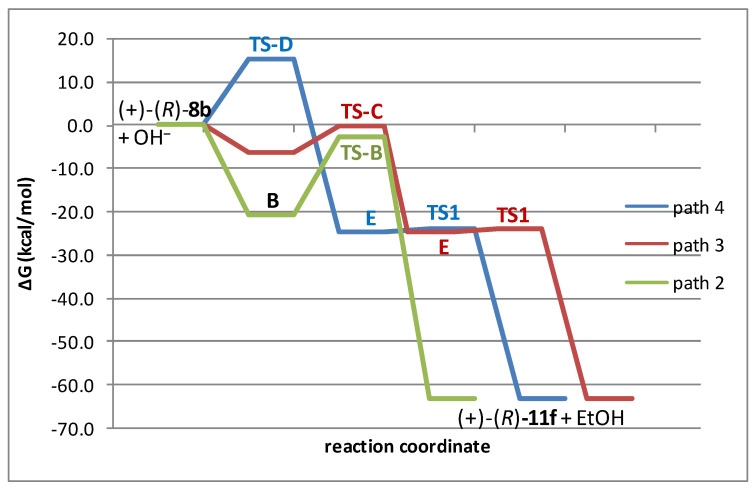
Gibbs free energy profiles for reaction paths resulting in the evolution of EtOH—2 (green), 3 (red) and 4 (blue); the profiles for the evolution of MeOH are very similar and are omitted for clarity.

Path 3 assumes the attack of the OH^(−)^ opposite the ^i^Pr group, with an initial formation of the high energy structure **C** with apical ^i^Pr and OH groups and equatorial alkoxy groups. Departure from the equatorial position is accompanied by a high energy barrier (see discussion of path 1)—so, another possibility is pseudorotation (**TS-C**), which gives a structure with alkoxy groups in the apical positions €. The **E** structure is the most stable among the sulfurane structures considered. Scheme 10 shows two isoenergetic conformers of **E**, differing in the orientation of the OH bond, which interacts with the alkoxy oxygen to facilitate the departure of the OMe or OEt groups, respectively. The transition states **TS1** and **TS2**—leading to the elimination of EtOH or MeOH, respectively—are, by only about 1 kcal/mol, higher in free energy than the corresponding **E1** and **E2** sulfuranes. However, the transition state for Berry pseudorotation (**TS-C**) is also relatively high in energy (Figure 5).

Path 4 involves the attack of the OH^(−)^ opposite the lone pair at the sulfur. This attack leads to a high energy transition state **TS-D**, which rearranges to **E** and finally decomposes with the evolution of ROH—as in path 3. However, the barrier associated with the formation of **TS-D** is so high that it prevents the reaction from proceeding along this pathway. The free energy profiles for the discussed paths 2–4 (for the case where (+)-(*R*)-**11f** + EtOH are produced) are presented in Figure 5. The corresponding profiles for the pathways leading to (−)-(*S*)-**11b** + MeOH are almost the same. The relative energies of the discussed isomeric sulfuranes and transition states are shown in Table 3.

Based on the DFT calculations, it can be concluded that path 1, involving the recombination of ions with the simultaneous formation of sulfuranes **A** and **B** (according to the A–E mechanism) followed by the dissociation of an alcohol molecule (MeOH or EtOH), is the most thermodynamically favorable. The other paths proceed through high energy transition states and are therefore less feasible.

**Scheme 11:**
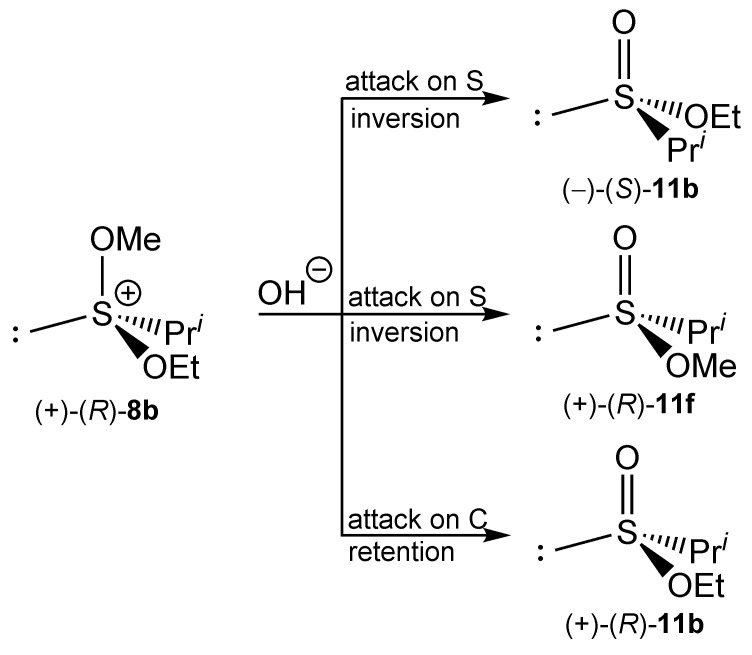
Bidirectional attack of the hydroxyl anion on sulfonium salt **8b** and its stereochemical consequences.

In view of this important statement, the reason why both the hydrolysis products—methyl and ethyl sulfinates **11**—are highly racemized, must be due to the other side-processes that accompany the substitution reaction. For a long time, it has been known that the alkoxysulfonium salts R_2_S^+^-OR behave as ambident electrophiles [19]. Depending on the nature of a nucleophile, one observes a nucleophilic attack not only on sulfur ions but also on the carbons of alkoxy groups, resulting in de-alkyation. In the case of dialkoxysulfonium salts **8**, which are more reactive than their monoalkoxy analogues, the bidirectional attack of the hydroxyl anion has an influence on the stereochemical outcome of the substitution reaction, as well as on the proportions of the two **11** sulfinates formed (see Scheme 11). Whereas the attack of a hydroxyl anion on the sulfur results in the formation of the methyl and ethyl sulfinates **11f** and **11b**—with the inversion of their configuration at S—the demethylation reaction (the attack of the OH^−^ at the methoxy carbon) leads to ethyl sulfinate **11b**, with a retained configuration at the sulfur.

As the hydrolysis reaction of the methoxy(alkoxy)sulfonium salts **8** proceeds under basic conditions and as the departing methoxy and alkoxy anions are present in the reaction mixture, a high extent of racemization of both the produced sulfinates (methyl sulfinate **11f** and alkyl sulfinates **11A–E**) may also be due to the symmetric exchange reactions at the chiral sulfur in the **11** esters (MeO/MeO, RO/RO). The racemization of the sulfinate **11f** is schematically depicted in Equation (2):

MeO^−^ + (+)-Pr*^i^*S(O)OMe ⇌ [MeO-Pr*^i^*S(O)-OMe]^−^ ⇌ (±)-(MeO)Pr*^i^*S=O + MeO^−^ (2)

More information on this type of identity substitution reactions at S and P may be found in our recent paper [9].

## 3. Materials and Methods

### 3.1. General Experimental Details

Commercial-grade reagents and solvents were used without further purification. THF and diethyl ether were distilled over NaH/benzophenone prior to use. Column chromatography was performed using a silica gel (70–230 mesh). The routine ^1^H NMR spectra were recorded at 60 and 90 MHz unless stated otherwise. Chemical shifts are quoted in parts per million (ppm) and reported relative to TMS as an internal standard. Boiling and melting points are uncorrected.

### 3.2. Synthetic Procedures

#### 3.2.1. Asymmetric Synthesis of Alkyl Isopropanesulfinates **11a–f**-General Procedure

To a solution of isopropanesulfinyl chloride (0.01 mol) in ether (50 mL) cooled to −70 °C, a mixture of proper alcohol (0.01 mol) and (−)-*N*,*N*-dimethyl-α-phenylethylamine (0.09 mol) dissolved in ether (40 mL) was added. The reaction mixture was stirred for 1 h. When the temperature of the reaction solution reached 20 °C, the etheral solution was washed with water (1 × 30 mL), diluted with 3% hydrochloric acid (1 × 30 mL) and with a solution (3%) of sodium hydrogen carbonate (1 × 10 mL). The reaction solution was then dried over MgSO_4_ and the ether was evaporated under reduced pressure. The sulfinate **11** was purified by chromatography (silica gel; eluent: *n*-hexane/ethyl ether, 4:1). The values for the optical rotation and optical purity of the sulfinates **11**, prepared as above, are given in Table 2.

**Pr*^i^*S(O)OCD_3_ 11a**: ^1^H NMR (CDCl_3_, 60 MHz): δ = 1.15 (d, 6H, (CH_3_)_2_CH, *J* = 6 Hz); 2.70 (sp, 1H, (CH_3_)_2_CH, *J* = 6 Hz); IR (film) 1130 cm^−1^ (υ S=O); n_D_^20^ = 1.4356.

**Pr*^i^*S(O)OEt 11b**: ^1^H NMR (CDCl_3_, 60 MHz): δ = 1.10 (d, 6H, (CH_3_)_2_CH, *J* = 6.5 Hz); 1.30 (t, 3H, CH_3_CH_2_O, *J* = 6.5 Hz); 2.70 (sp, 1 H, (CH_3_)_2_CH, *J* = 6.5 Hz); 4.00 (q, 2H, CH_3_CH_2_O, *J* = 6.5 Hz); IR (film) 1125 cm^−1^ (υ S=O); n_D_^20^ = 1.4350.

**Pr*^i^*S(O)OPr*^n^* 11c**: ^1^H NMR (CDCl_3_, 60 MHz): δ = 1.05 (t, 3H, CH_3_(CH_2_)_2_O, *J* = 6 Hz); 1.15 (d, 6H, (CH_3_)_2_CH, *J* = 6 Hz); 1.70 (sx, 2H, CH_3_CH_2_CH_2_O, *J* = 6 Hz); 2.70 (sp, 1H, (CH_3_)_2_CH, *J* = 6 Hz); 3.85 (t, 2H, CH_3_CH_2_CH_2_O, *J* = 6 Hz); IR (film) 1130 cm^−1^ (υ S=O); n_D_^21^ = 1.4400.

**Pr*^i^*S(O)OBu*^n^* 11d**: ^1^H NMR (CDCl_3_, 60 MHz): δ = 0.95 (t, 3H, CH_3_(CH_2_)_3_O, *J* = 6 Hz); 1.10 (d, 6H, (CH_3_)_2_CH, *J* = 6 Hz); 1.10–1.80 (m, 4H, CH_3_(CH_2_)_2_CH_2_O, *J* = 6 Hz); 2.70 (sp, 1H, (CH_3_)_2_CH, *J* = 6 Hz); 3.90 (t, 2H, CH_3_CH_2_CH_2_O, *J* = 6 Hz); IR (film) 1135 cm^−1^ (υ S=O); n_D_^20^ = 1.4415.

**Pr*^i^*S(O)ONp 11e**: ^1^H NMR (CDCl_3_, 60 MHz): δ = 0.90 (s, 9H, (CH_3_)_3_CH); 1.10 (d, 6H, (CH_3_)_3_CH, *J* = 6 Hz); 2.70 (sp, 1H, (CH_3_)_2_CH, *J* = 6 Hz); 3.60 (s, 2H, (CH_3_)_3_CCH_2_O); IR (film) 1125 cm^−1^ (υ S=O); n_D_^21^ = 1.4430.

**Pr*^i^*S(O)OMe 11f**: ^1^H NMR (CDCl_3_, 60 MHz): δ = 1.15 (d, 6H, (CH_3_)_2_CH, *J* = 6 Hz); 2.70 (sp, 1H, (CH_3_)_2_CH, *J* = 6 Hz); 3.80 (s, 3H, CH_3_O); IR (film) 1128 cm^−1^ (υ S=O); n_D_^20^ = 1.4395.

Elemental analysis data were obtained with an accuracy of C = ±0.34%; H = ±0.28%; S = ±0.60%.

#### 3.2.2. Synthesis of Optically Active Alkoxy(methoxy)isopropyl Sulfonium Trifluoromethanesulfonates **8A–E**-General Procedure

To a stirred solution of alkyl isopropanesulfinate **11** (0.001 mol) in nitromethane (20 mL), methyl trifluoromethanesulfonate (0.002 mol) was added. After 30 min, nitromethane and an excess of methylating agent were removed under reduced pressure at a temperature below 30 °C to obtain the sulfonium salts **8**. Owing to their limited stability, the sulfonium salts **8** so-obtained were quickly characterized by ^1^H NMR spectroscopy (see below) and subjected to alkaline hydrolysis.

**CD_3_O(MeO)Pr*^i^*S^+^ 8a**: ^1^H NMR (CD_3_NO_2_, 60 MHz): δ = 1.25 (d, 6H, (CH_3_)_2_CH, *J* = 6 Hz); 3.20 (sp, 1H, (CH_3_)_2_CH, *J* = 6 Hz); 4.20 (s, 3H, CH_3_O).

**EtO(MeO)Pr*^i^*S^+^ 8b:** ^1^H NMR (CD_3_NO_2_, 60 MHz): δ = 1.25 (d, 6H, (CH_3_)_2_CH, *J* = 6 Hz); 1.40 (t, 3H, CH_3_CH_2_O, *J* = 6Hz); 3.20 (sp, 1H, (CH_3_)_2_CH, *J* = 6 Hz); 4.25 (s, 3H, CH_3_O); 4.65 (q, 2H, CH_3_CH_2_O, *J* = 6Hz).

**Pr*^n^*O(MeO)Pr*^i^*S^+^ 8c:** ^1^H NMR (CD_3_NO_2_, 60 MHz): δ = 1.15 (t, 3H, CH_3_(CH_2_)O, *J* = 6 Hz); 1.35 (d, 6H, (CH_3_)_2_CH, *J* = 6Hz); 1.85 (sx, 2H, CH_3_CH_2_CH_2_O, *J* = 6Hz); 3.20 (sp, 1H, (CH_3_)_2_CH, *J* = 6 Hz); 4.20 (s, 3H, CH_3_O); 4.45 (t, 2H, CH_3_CH_2_CH_2_O, *J* = 6Hz).

**Bu*^n^*O(MeO)Pr*^i^*S^+^ 8d:** ^1^H NMR (CD_3_NO_2_, 60 MHz): δ = 1.10 (t, 3H, CH_3_(CH_2_)O, *J* = 6 Hz); 1.30 (d, 6H, (CH_3_)_2_CH, *J* = 6Hz); 1.30–2.05 (m, 4H, CH_3_CH_2_CH_2_CH_2_O, *J* = 6Hz); 3.20 (sp, 1H, (CH_3_)_2_CH, *J* = 6 Hz); 4.25 (s, 3H, CH_3_O); 4.60 (t, 2H, CH_3_(CH_2_)CH_2_O, *J* = 6Hz).

**NpO(MeO)Pr*^i^*S^+^ 8e:** ^1^H NMR (CD_3_NO_2_, 60 MHz): δ = 1.05 (s, 9H, (CH_3_)_3_CCH_2_); 1.25 (d, 6H, (CH_3_)_2_CH, *J* = 6Hz); 3.20 (sp, 1H, (CH_3_)_2_CH, *J* = 6 Hz); 4.25 (s, 3H, CH_3_O); 4.35 (s, 2H, (CH_3_)_3_CCH_2_O).

#### 3.2.3. Alkaline hydrolysis of Alkoxy(methoxy)isopropyl Sulfonium Trifluoromethanesulfonates **8A–E**-General Procedure

The sulfonium salts **8A–E** prepared as described above were quickly dissolved in water (10 mL) and treated with a 0.02 N solution of sodium hydroxide in the presence of phenolphthalein. When hydrolysis was complete (yellowish color of the reaction solution), the water phase was extracted with *n*-hexane (3 × 10 mL) and chloroform (2 × 10 mL). The combined organic solutions were dried over MgSO_4_ and evaporated under reduced pressure. The hydrolysis products (two sulfinate esters **11**) were separated by preparative gas chromatography. Their optical rotations and proportions are summarized in Table 1 and Table 2.

### 3.3. Theoretical Methods

All quantum mechanical calculations were performed using the Gaussian 16 suite of programs [20]. The geometries of the model compounds were optimized using two DFT methods: the hybrid B3LYP density functional model [21] corrected for dispersion interactions using the Grimme D3(0) empirical term [22], with the Def2-TZVP basis set [23] was used in aqueous solutions; SCRF calculations in water were performed using the CPCM model with UFF atomic radii, implemented in Gaussian 16 [20]. All stationary points were identified as stable minima or transition states by frequency calculations. Geometry optimizations were performed using normal convergence criteria as defined in Gaussian. The integration grid was set to ultrafine. The vibrational analysis provided thermal enthalpy and entropy corrections at 298 K within the rigid rotor/harmonic oscillator/ideal gas approximation [20]. RRHO cutoffs were not applied. Thermochemical corrections were scaled by a factor of 0.99 [24].

## 4. Conclusions

The present study was undertaken with two major aims: The first was to extend our investigations on nucleophilic substitution reactions at the sulfur atom to new model organosulfur structures containing two potential leaving groups—namely, optically active dialkoxysulfonium salts. The alkaline hydrolysis of these salts was selected as a simple model substitution reaction. The second task was to establish the stereochemistry and mechanism of the above reaction, as well as to establish its relative reaction energetics—especially in the formation and decomposition of the intermediates. The results obtained in the frame of this project are given below.

1. A series of optically active alkyl isopropanesulfinates **11** was prepared by asymmetric synthesis, developed in our laboratory;
2. The relatively stable alkoxy(methoxy)sulfonium salts **8** were obtained by methylation of the **11** sulfinates with methyl trifluoromethanesulfonate;
3. Alkaline hydrolysis of the sulfonium salts **8** afforded two **11** sulfinates, methyl isopropanesulfinate and alkyl isopropanesulfinate—both with a slightly prevailing inversion of their configuration at the sulfonium sulfur atom;
4. A high extent of racemization of the sulfinate products is due to competitive side-reactions, with the attack of the hydroxy anion at the carbon atom of the methoxy group and the identity alkoxy exchange reactions in alkyl sulfinates being hydrolysis products;
5. DFT calculations revealed that alkaline hydrolysis of **8** salts occurs stepwise according to addition-elimination (A–E) mechanisms involving the formation of tetracoordinate intermediates with a trigonal bipiramidal structure;
6. The hydrolysis products, i.e., the methyl and alkyl sulfinates, were formed via the most stable sulfurane intermediates, with hydroxy and alkoxy substituents in the apical positions and their direct decomposition.

## Figures and Tables

**Table 1:** Selected data on alkyl sulfinates **11**, dialkoxysulfonium salts **8** and sulfinates **11** formed after alkaline hydrolysis of **8**.

(RO)Pr*^i^*S(O) (11a–e)				(RO)(MeO)Pr*^i^*S^+^ (8a–e)		(RO)Pr*^i^*S(O) (11a–e)		
R	[α]_D_	op	conf	[α]_D_	conf	[α]_D_	op	conf
a, CD_3_	+21.3°	18	*R*	+0.2° *	*R*	-	-	-
b, Et	+23.8°	20	*R*	+0.5°	*R*	−3.1°	2.6	*S*
c, *^n^*Pr	+35.1°	27	*R*	+1.9°	*R*	−1.4°	2.7	*S*
d, *^n^*Bu	+24.6°	20	*R*	+0.2°	*R*	−1.2°	1	*S*
e, Np	+17.9°	15	*R*	+0.84°	*R*	−3.5°	3	*S*

* determined at 365 nm.

**Table 2:** Content of methyl isopropanesulfinate **11f** in a mixture with alkyl isopropanesulfinates **11a–e**.

Sulfonium Salt (8a–e)	iPrS(O)OMe (11f) (%)	
	^1^H NMR	G-C
(**a**) MeO(CD_3_O)Pr*^i^*S^+^	40	44
(**b**) EtO(MeO)Pr*^i^*S^+^	18	15
(**c**) iPrO(MeO)Pr*^i^*S^+^	10	9
(**d**) nBuO(MeO)Pr*^i^*S^+^	2	3
(**e**) NPO(MeO)Pr*^i^*S^+^	0	0

**Table 3:** Relative enthalpies and Gibbs free energies (kcal/mol) of sulfuranes (Scheme 2; ΔH_r_, ΔG_r_—relative to the sum of the free energies of the substrates ((EtO)iPrSOMe^(+)^ + OH^(−)^), ΔH_rel_, ΔG_rel_—relative to the most stable sulfurane isomer (**E2**)) in water.

Sulfurane S^IV^	ΔH_r_	ΔG_r_	ΔH_rel_	ΔG_rel_
(EtO)Pr^i^S(OMe)OH, HO-S-OMe-ax (**A**)	−31.4	−21.7	3.7	2.9
(EtO)Pr^i^S(OMe)OH, HO-S-OEt-ax (**B**)	−32.0	−20.7	3.1	3.9
(EtO)Pr^i^S(OMe)OH, HO-S-iPr-ax (**C**)	−16.5	−6.3	18.6	18.3
(EtO)Pr^i^S(OMe)OH, HO-S-:-ax (**D**) (not found)	NA	NA	NA	NA
(EtO)Pr^i^S(OMe)OH, EtO-S-OMe-ax, OH^…^Me (**E1**)	−34.9	−24.6	0.2	0
(EtO)Pr^i^S(OMe)OH, EtO-S-OMe-ax, OH^…^OEt (**E2**)	−35.1	−24.6	0	0
(EtO)Pr^i^S(OMe)OH, EtO-S-OMe-ax, leaving OMe (**TS1**)	−34.0	−23.3	0.9 *	1.3 *
(EtO)Pr^i^S(OMe)OH, EtO-S-OMe-ax, leaving OEt (**TS2**)	−34.5	−24.0	0.6	0.6
(EtO)*^i^*PrS(OMe)OH, HO-S-OMe-ax, leaving OEt (**TS1**(**eq**))	−0.7	9.8		
(EtO)*^i^*PrS(OMe)OH, HO-S-OEt-ax, leaving OMe (**TS2**(**eq**))	−1.1	9.2		
(EtO)Pr^i^S(OMe)OH, HO-S-:-ax (**TS-D**)	5.1	15.4	40.2	40.2
Pseudorotation (HO,Me)→(Pr^i^,OEt) (**TS-B**)	−14.6	−2.6		
Pseudorotation (HO,Pr^i^)→(MeO,OEt) (**TS-C**)	−12.7	−0.1		

* energies calculated relative to **E1**, as the reaction precursor.

## Data Availability

Data is contained within the article.

## Sample Availability

Samples of the compounds are not available from the authors.
